# Parameters of oxidative stress variation depending on the concentration of inorganic zinc compounds

**Published:** 2015

**Authors:** R Grigorescu, MI Gruia, V Nacea, C Nitu

**Affiliations:** * ”Carol Davila” University of Medicine and Pharmacy, Bucharest, Romania; ** ”Prof. Dr. Alex. Trestioreanu” Oncological Institute, Bucharest, Romaniaa

**Keywords:** antioxidant, ZnSO4, ZnCl2, ZnBr2

## Abstract

Zinc deficiency is a problem faced by a large number of people, a U.S. study showing that only 46% of the population aged over 71 years has the best amount of zinc in the body. Due to the very complex role of zinc deficiency in this trace, it can occur through a variety of symptoms affecting multiple body functions.

Zinc was demonstrated to have the ability to neutralize free radicals protecting the body from the harmful effects of these effects, ultimately leading to atherosclerosis and cardiovascular disease derived from premature aging, the immune and immune disorders and increased risk of cancer.

The purpose of the paper is to identify the role of antioxidant systems, with Zn2+ ions in the center of defense and decrease oxidative stress in dynamic interaction with malignant transformed cells.

## Introduction

The role of zinc in the body is very complex given the multitude of enzymes that it contains. It is mainly responsible for growth, wound healing and regulation of testosterone metabolism and the proper functioning of immunity. In adult human body, there are 2-3 grams of zinc entering the enzyme system, structure more bone and skin [**1**].

The daily intake of zinc in the human body is of 10-15 mg/ day [**1**].

After migration, foods that contain zinc are absorbed in the duodenum and jejunum. Factors that interfere with zinc absorption would probably belong to the prostaglandin E group and a low molecular weight metalloproteinases, metallothionein [**1**,**3**]. Zinc absorption is diminished by the presence of copper in interstitial lumen due to the competition for occupying the same plasma albumin coordination points. Also zinc absorption is hampered by the presence of large amounts of calcium, phytic acid complexes of amino acids or carbohydrates. After absorption, Zn 2 + is transported as complex mucosal cells and subsequently transferred to serum albumin [**2**,**5**,**8**].

Like calcium, serum zinc is in the unionized (34%) form, strongly bound to plasma proteins and ionized (66%) bound to labile protein form that is transported [**2**,**5**,**8**].

Zinc deficiency is a problem faced by a large number of people, a U.S. study showing that only 46% of the population aged over 71 years has the best amount of zinc in the body [**4**,**6**,**12**].

Due to the very complex role of zinc deficiency in this trace, it can occur through a variety of symptoms affecting multiple body functions.

Zinc plays an important role in horse protection against free radicals actions expressed directly by binding to free radicals, zinc acts as an antioxidant and anti-inflammatory agent [**7**,**9**,**10**]. Zinc was demonstrated to have the ability to neutralize free radicals protecting the body from the harmful effects of these effects ultimately leading to atherosclerosis and cardiovascular disease derived them from premature aging, the immune and immune disorders and increased risk of cancer [**13**,**15**,**16**].

Involvement of zinc in antioxidant stress reduction, stress antioxidant role in the development of malignant tumors, involvement of zinc in apoptosis and faulty functioning correlation between apoptosis and cancer have led us to realize the experiments reported in the present paper [**11**,**14**].

The purpose of the paper was to identify the role of antioxidant systems, with Zn2+ ions in the center of defense and decrease oxidative stress in dynamic interaction with malignant transformed cells. 

## Materials and methods

In order to identify the effect of non-enzymatic inorganic antioxidants depending on concentration measurements, the parameters of oxidative stress, namely lipid peroxides, antioxidants albumin and total thiol groups were made in an ex-vivo experimental model in which the variation depending on the time of action and the concentration of inorganic compounds containing zinc on cells from ascites tumor-bearing animals Walker 256 were followed.

What should also be mentioned is that this experimental tumor can be maintained by serial passage without modifying the properties; in addition, it can be induced by ascites cells. They will initiate inoculated solid tumors which, in turn, after the 21st day of the implant, can cause intra-abdominal ascites fluid. This fluid contains ascites tumor cells identical to those initiatives.

Ascites fluid was collected and incubated for 30 minutes with the three compounds investigated, namely ZnSO4, ZnCl2 and ZnBr2 in the following concentrations: 1g/ ml, 100mg/ ml, 10 mg/ ml, 1 mg/ ml. The results were reported to two witnesses, namely a blank compound that did not contain zinc was replaced with distilled water and the second witness containing tumor cells without added compound antioxidant potential.

## Results and discussion

**Fig. 1:**
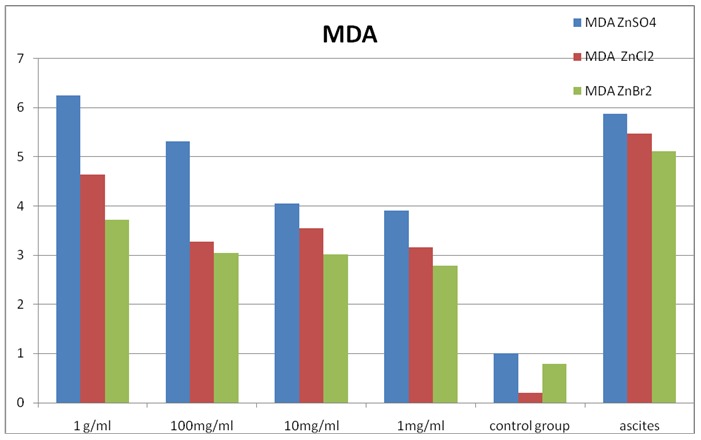
Determination of lipid peroxidation reaction in sea squirt cellules incubated with inorganic zinc

The first reaction was the determination of lipid peroxidation, starting from the premise that lipid molecules are first attacked by free radicals and most easily affected by chain reactions initiated by reactive oxygen species. The results are presented in **Fig. 1** and clearly suggest that tumor cells show intense oxidative stress, measured reaction ascites cells have higher values with small changes and many insignificant data errors. Antioxidant activity is dependent on the concentration, but contrary to our expectations, the effect was inversely proportional to the concentration, the lowest values were recorded at low concentrations thus suggesting that these compounds are effective in this experimental model in low concentrations and over a certain amount they are pro-oxidants.

A second test reaction followed the oxidative attack on proteins and albumin in particular, determining the release in the reaction of total thiol groups, resulting from destructive attack/ protein oxidative reaction medium.

**Fig. 2:**
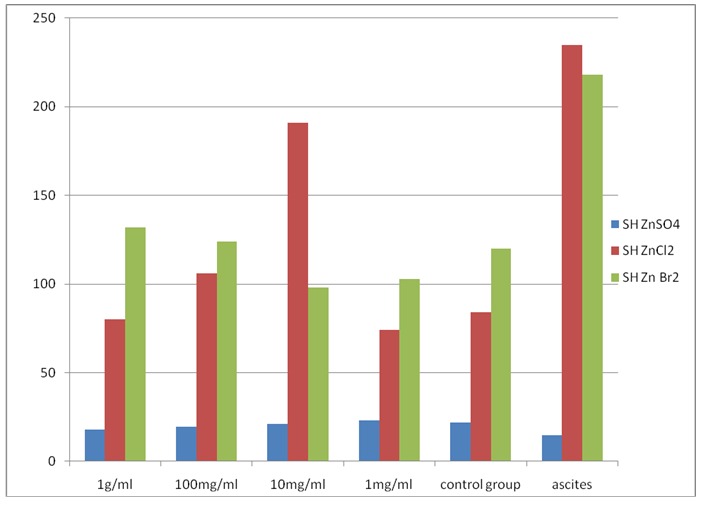
Determination of total thiols cellule ascites albumin treated with zinc

The results are different depending on the compounds tested, namely zinc sulfate has no influence on the effect of protein degradation, the values recorded are approximately constant, zinc chloride and bromide interfere with antioxidant function of concentration. The curves obtained (**Fig. 2**) showed the production of thiol reducing the effective albumin concentration in the range of 1g/ ml - 100 mg/ ml and 10mg/ ml chloride - 1mg/ ml for bromide. In addition, from these results, it resulted a balance of the ability Antioxidant/ pro-oxidant function of the test compound concentration.

**Fig. 3:**
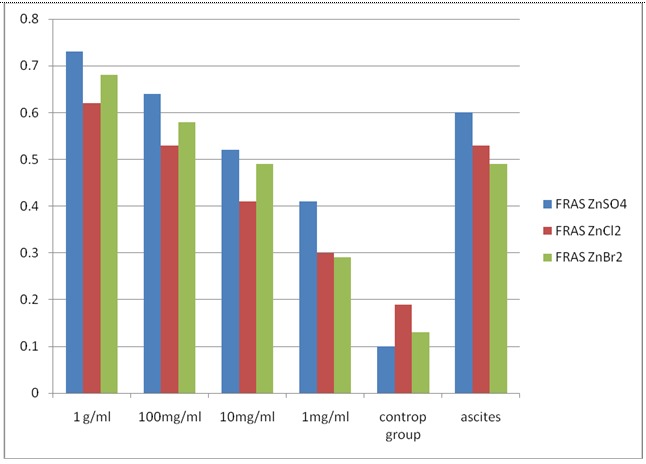
The monitoring of reduction reaction of iron on inorganic zinc compounds

The third parameter investigated refers to the ability to reduce the iron compound and the reaction is used to track the total antioxidant level, modulated by the three compounds. Recorded profile was shown in the chart below (**Fig. 3**) and suggested lower values depending on the concentration. The most effective action in all cases was given in the lowest concentration, contrary to our expectations.

Recorded data were consistent with the literature. Here there are many controversies related to the use of antioxidants and especially in patients with cancer.

Following the experimental data obtained, we could say with certainty that non-enzymatic antioxidants act inorganically. Depending on the concentration, they are more efficient in low concentrations than those grown. This could be explained by the fact that they may be due to their low molecular weight molecules that signal to initiate a complete response of all endogenous antioxidant systems fight. If exceeded, the balance that kept oxidative stress normal, registered pro-oxidant reactions.

Extrapolating, the results obtained from tumor-bearing patients and especially those undergoing treatment or chemical or radiological oncostatics must be used with great caution antioxidants. Thus, radiotherapy based on free radical production may be compromised if administered during experimental irradiation exogenous antioxidants, they will be effective at the end of radiotherapy with radical left end capture species away from the workplace. The same principles should be applied in the case of chemotherapy, especially when taken with chemotherapy thrown anthracycline, whose local action is to induce cytotoxic reactive oxygen species. Effect can be minimized by exogenous antioxidants during treatment; they might be prescribed and will be more effective after the treatment courses.

## Discussion

It can be said with certainty that the three inorganic compounds containing zinc (Zn SO4, ZnCl2, ZnBr2) have an antioxidant effect depending of concentration. They are more effective at low concentrations, suggesting a very high caution in prescribing them because they can easily turn into pro-oxidants, especially in the presence of tumor cells that augmented antioxidant protection systems from normal cells.
